# The effectiveness of physiotherapy interventions on pain and quality of life in adults with persistent post-surgical pain compared to usual care: A systematic review

**DOI:** 10.1371/journal.pone.0226227

**Published:** 2019-12-13

**Authors:** Aleisha Robinson, Jenna McIntosh, Hamish Peberdy, David Wishart, Georgia Brown, Henry Pope, Saravana Kumar

**Affiliations:** University of South Australia, Adelaide, South Australia, Australia; University of Mississippi Medical Center, UNITED STATES

## Abstract

**Background:**

Persistent post-surgical pain (PPSP) is a common condition following surgery, resulting in persistent pain and reduced quality of life (QoL). While pharmacological management is common, its effectiveness remains equivocal. This systematic review investigated the effectiveness of physiotherapy management in adults with PPSP in comparison to usual care.

**Methods:**

A systematic search of six electronic databases was conducted. Studies of human adults (>18 years) with PPSP localised or directly referred from the surgical site, pain persisting for at least two months post-surgery and with physiotherapy as the intervention were included. This review was conducted and reported in line with the Preferred Reporting Items for Systematic Reviews and Meta-Analyses (PRISMA) statement. The McMaster critical review form for quantitative studies was utilised to assess the methodological quality. A descriptive synthesis was undertaken due to the heterogeneity of the included studies.

**Results:**

Of the 1395 articles that were screened, eight studies met the inclusion criteria. A diverse range of physiotherapy interventions were utilised, and effectiveness was measured through diverse outcomes and measures. Summarised findings from the heterogenous evidence base indicated that physiotherapy interventions for PPSP has a positive impact across a range of outcomes, including pain, quality of life (QoL), physical function and depression. While these are encouraging findings, the current evidence base lacks uniformity with regards to participant characteristics, time periods since diagnosis, interventions delivered, and its parameters, and outcomes measured.

**Conclusion:**

Due to ongoing challenges in the management of PPSP, alternate treatment strategies such as physiotherapy are being trialled. Despite a number of methodological constraints, current evidence indicates that physiotherapy could play a role in the management of PPSP.

**PROSPERO registration:**

CRD42019129580

## Introduction

Persistent post-surgical pain (PPSP), also known as chronic postsurgical pain (CPSP) [1–2], is a common condition that is evident following surgeries of varying complexities [3–4]. Kehlet, Jensen and Woolf [5] suggest that acute post-surgical pain becomes persistent in 10–50% of patients, with 2–10% experiencing severe pain. PPSP is a major economic burden in western societies, with direct and indirect costs estimated to be almost double the cost of cardiovascular diseases or cancer [6]. The current and universally agreed definition of PPSP has four distinct components: persistent pain at three months following surgery (however, it can also range from two to six months) [7]; pain-free before surgery or any preoperative pain must have varying characteristics or increased intensity; pain must be localised or directly referred from the surgical site; exclusion of pain caused by other factors, such as, infection or malignancy [4,7].

Although the underlying cause and pathophysiology of PPSP is poorly understood, its aetiology is complex and thought to be multifactorial; involving a neural component and pre-, intra- and post-surgical factors [8–10]. A patient’s psychosocial wellbeing has been suggested to play a major role in the development of PPSP [10–12]; a study by Geiss and colleagues [13] proposed that chronically stressed patients are more likely to have poorer surgical outcomes. In addition, evidence suggests higher levels of anxiety and depression can exacerbate pain, possibly through a dysregulation of immune responses causing a pro-inflammatory state and thus making a patient more susceptible to pain [14–15]. Other common risk factors include genetic predisposition, intraoperative nerve damage and severity and duration of acute post-surgical pain [3,11].

In the acute stages post-surgery, the predominant pain type is nociceptive; however, in the chronic stages this pain becomes complex and may involve nociceptive, neuropathic, central and inflammatory components [5]. Neuropathic pain has been reported as a major contributor to PPSP, which is likely due to damage to the nervous system resulting in sensory loss and hypersensitivity [4–5]. In contrast to this, Guastella and colleagues [16] investigated the neuropathic components in PPSP following thoracotomy and reported only 29% of participants with neuropathic pain, suggesting two groups of PPSP: neuropathic PPSP and non-neuropathic PPSP. Inflammatory pain occurs with tissue injury as well as inflammation and is often associated with acute pain; if however, the inflammatory response continues, this pain is maintained [5]. Finally, central sensitisation, which is the synaptic plasticity that may occur following signaling from noxious peripheral stimuli, could play a role by ultimately causing abnormal responses to a normal sensory input [5].

Currently, primary management for PPSP centres on pharmaceutical and medical interventions, despite the poorly understood pathophysiology [17–19]. A recent systematic review by Wylde and colleagues [17] investigating the management of PPSP, was unable to conclude the effectiveness of any modality, pharmaceutical or medical, to reduce pain in PPSP patients. Additionally, a systematic review by Chaparro and colleagues [18] investigated different pharmaceutical approaches to prevent the occurrence of PPSP. They found the incidence of chronic pain post-surgery was reduced with ketamine administration; however, multiple other pharmaceutical approaches showed no reduction [18]. Andreae and Andreae [19] investigated the effectiveness of anaesthetic and observed minor pain reduction in PPSP following thoracotomy and mastectomy. Medical interventions are currently accepted for management of PPSP, despite ambiguous evidence to support its use [20].

Some non-pharmacological interventions have also been utilised in the management of PPSP, including psychotherapy, acupuncture, osteopathy and physiotherapy [21–23]. Emerging studies investigating physiotherapy management are shown to have some effectiveness in reducing pain, improving QoL, physical functioning and depression [22–29]. However, there is a limited evidence base; and to date, no systematic reviews have been published evaluating the effectiveness of physiotherapy for PPSP. Therefore, the present review aims to evaluate the effectiveness of physiotherapy interventions as compared to usual care on pain and quality of life in PPSP subjects.

## Methods

### Search protocol and registration

The protocol for this systematic review was registered with the international prospective register of systematic reviews–PROSPERO (Registration # CRD42019129580).

### Search strategy

This review was conducted and reported in line with the Preferred Reporting Items for Systematic Reviews and Meta-Analyses (PRISMA) statement and correlates with the PRSIMA checklist (S1 Appendix). The PICO format was utilised in the development search strategy with search terms and limits relating to PPSP (population of interest) and physiotherapy (intervention). The development of the search strategy was informed by discussions with a University of South Australia Academic Librarian. As means of trialling the search strategy, two reviewers (HP and GB) searched a database independently. The results from the search was then cross-referenced to ensure there was consistency in the search process. If the outcomes of the search results were different, the two reviewers met to discuss these differences and conflicts were resolved through discussion. Only after consistency in the search process was achieved, the reviewers commenced formal searching of the databases. A comprehensive search was developed, and six electronic databases were searched between 26^th^-28^th^ of March 2019. These databases include: Ovid Medline, Ovid Embase, Ovid Emcare, Cochrane (all available dates), PEDro, PsycINFO. Grey literature was additionally searched for further relevant information. As no systematic reviews were previously published, no date restrictions were applied. The following search terms were used as MESH headings: physical therapy modalities, physiotherapy, postoperative pain, chronic pain. The full search strategy for a database is outlined in S3 Appendix and syntax in S4 Appendix. The reference lists of all included studies were reviewed to obtain any relevant studies that were not located by electronic search.

### Study designs

All types of primary quantitative study designs were eligible for inclusion, such as randomised controlled trials (RCTs), controlled clinical trials (CCTs), case studies, pre-post cohort, quasi-experimental studies. Secondary research designs were included for pearling purposes and qualitative research designs were excluded. The eligibility criteria for the population-intervention-comparator-outcome (PICO) is outlined below.

### Population

The studies were included if participants were adults (>18 years) of either gender who have PPSP. PPSP was defined by persistent pain from two months following [7]; pain-free before surgery or any preoperative pain must have varying characteristics or increased intensity; pain must be localised or directly referred from the surgical site; exclusion of pain caused by other factors, such as, infection or malignancy [4,7]. PPSP Studies were excluded if participants had undergone amputations (phantom limb pain), had pre-existing pain that had not changed or intensified since surgery, acute pain (less than two months post-surgery) or other causes of pain, such as infection or malignancy.

### Intervention

Studies were included if the physiotherapeutic intervention was prescribed, or could be performed, by a Physiotherapist (or an equivalent professional group such as physical therapist). Physiotherapeutic interventions included but were not limited to electrotherapy, exercise, education, manual therapy (manipulation, mobilisation, massage) etc. Studies were excluded if the intervention was self-prescribed by the patient without the direct involvement of a physiotherapist (such as topical creams or gels) or had a pharmaceutical focus. As physiotherapy is commonly provided as a package of care (comprising of multiple interventions), the review did not limit to a single intervention or set specific parameters of intervention.

### Comparator

The acceptable comparators were control (no intervention provided) or ‘usual care’ which typically focusses on pharmacological (non-opioid analgesics, opioids, and adjuvant analgesics) and other conservative interventions.

### Outcome

The search was not limited to any specific outcomes, due to the multidimensional nature of pain and the variety of outcomes related to the effects of physiotherapy interventions for pain. Outcomes of interest included but were not limited to: pain, QoL, depression, anxiety and activities of daily living.

### Literature search

Once the search strategy was developed, a review protocol was established (S2 Appendix), and databases were searched. Search results from each of the electronic databases were transported into the referencing management program, Endnote^TM^ to organise and sort identified studies. Duplicate results found during the search process were then deleted through Endnote^TM^. From the Endnote^TM^ library, all records were uploaded into Covidence^TM^, where studies were accepted or removed through screening the title and abstract against the PICO criteria. The subsequent relevant studies’ full texts were then independently analysed by two reviewers to determine their eligibility for the PICO criteria. Any disagreements were resolved by discussion or with a third reviewer, where required.

### Methodological quality

The included studies were independently reviewed and ranked using the ‘Intervention category’ of the National Health and Medical Research Council (NHMRC) hierarchy of evidence [30], by all six reviewers. Any disagreements were resolved by discussion or with the independent reviewer (SK), where required. A modified McMaster Critical Review Form for Quantitative Studies [31] was used following the guidelines to assess the methodological quality of the included studies, shown in appendix 2. This tool assessed eight main components: study purpose; literature review; study design (all experimental designs); sample (participants description, size justification, ethics and consent); outcomes (reliability and validity, outcome areas and measures used); intervention (description, contamination and co-intervention); results (statistical and clinical significance, analysis methods and drop outs) and conclusion with implications to practice (limitations and biases). The individual components were rated as ‘yes’, ‘no’, ‘not addressed’ or ‘NA-Not Applicable’. A score of ‘1’ was given to ‘yes’, ‘0’ to ‘no and not-addressed'; if ‘NA’ category applied then the total scoring was changed accordingly. Dependent on the research design and applicable components, the maximum total score of a study was 14. Each study was independently rated by each reviewer and any disagreements were resolved by discussion or with independent reviewer (SK), where required.

### Data management

A customised data extraction was developed by the reviewers on Microsoft Excel^TM^ Spreadsheets, including information related to: study design, participants’ information (age, sex, diagnosis), intervention components, intervention duration, dose and frequency of intervention, experimental design, method of randomisation, outcome measures, statistical analysis and results of pre- and post-analysis or other appropriate results. All six reviewers independently extracted data using this tool to ensure reliability and consistency. Any disagreement was resolved through discussion and consultation with another reviewer (SK), where needed.

### Synthesis of results

Due to heterogeneity in the included studies, a meta-analysis was not conducted. Instead, a descriptive synthesis of results was undertaken, following the NHMRC FORM framework [32]. This framework has been widely used in previous systematic reviews successfully [33–35]. The framework consists of five main components: 1. Quantity and quality of evidence; 2. consistency; 3. clinical impact; 4. generalisability; and 5. applicability to the Australian health care setting. The final component was not used in the present systematic review due to its geographical focus.

## Results

### Study selection

**Fig 1:**
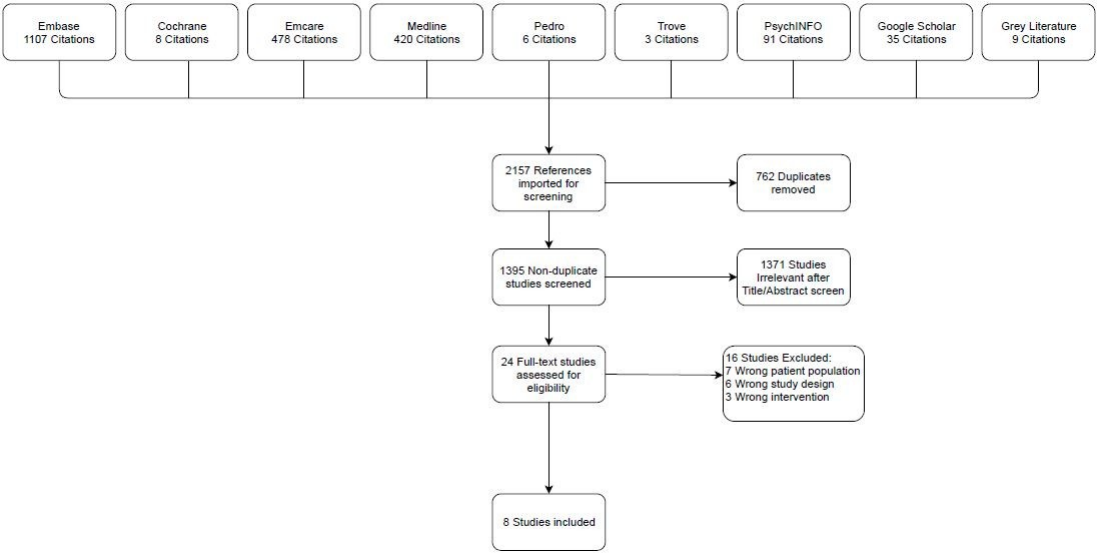
PRISMA flowchart for included studies.

The literature search returned 2157 total results, 762 duplicates were removed. The title and abstract of 1395 articles were screened following pooling of searches and of 1371 irrelevant articles were removed. 24 studies were reviewed in full, of which 16 studies were excluded: seven due to the study population not matching the aforementioned inclusion/ exclusion criteria; six for the incorrect study design and three for the incorrect intervention. Eight studies successfully met the eligibility criteria and were included in this study. The PRISMA flowchart is outlined in Fig 1.

### Risk of bias

Table 1 shows an overview of the NHMRC levels of evidence and allocated scores based on the McMaster Quantitative Critical Appraisal Tool [31] of the eight included studies. The study designs comprised of one RCT [24], one CCT [25] one pre-post study [22], three case series [23, 26–27] and two case reports [28–29]. The two highest critical appraisal scores, 100% and 91.6% were allocated respectively to the studies by Nunez-Cortez [26] and Albayrak [25] and colleagues. The lowest score was allocated to Vas and colleagues with 55.5%, suggesting the lowest level of study quality out of the eight included studies. Overall, what was done well amongst seven of the included studies were the discussion of relevant background information and justifying the research [22–27,29]. In addition, all studies used valid and reliable outcome measures [22–29].

**Table 1:** Levels of evidence and modified McMaster results of methodological quality.

Study	Study Design	NHMRC Level	Items on Modified McMaster critical review form																Raw Score	
			1	2	3	4a	4b	4c	5a	5b	6a	6b	6c	7a	7b	7c	7d	8		%
Albayrak [25]	Non-Randomised Controlled Trial	Level III-2	Y	Y		39	Y	Y	Y	Y	Y	NAD	NAD	Y	Y	N	Y	Y	11 out of 12	91.60%
Bailey [22]	Pre-post	Level III-3	Y	Y		43	Y	N	Y	Y	Y	N/A	N	Y	Y	Y	Y	Y	11 out of 13	84.6%
Bjersa [23]	Case Series (report)	Level IV	Y	Y		8	Y	Y	Y	Y	N	N/A	NAD	N	Y	Y	Y	Y	10 out of 12	83.3%
Nunez-Cortes [26]	Case series (report)	Level IV	Y	Y		14	Y	Y	Y	Y	Y	N/A	NAD	Y	Y	Y	Y	Y	12 out of 12	100%
Pfitser [24]	Randomised Controlled Trial	Level II	Y	Y		58	Y	Y	Y	Y	N	NAD	N	Y	Y	Y	Y	Y	11 out of 13	84.6%
Pons [28]	Case Report	Level IV	Y	Y		1	N	N/A	Y	Y	Y	N/A	NAD	N	Y	N	N/A	Y	7 out of 10	70%
Vas [27]	Case Series (report)	Level IV	N	N		2	Y	N/A	Y	Y	Y	N/A	NAD	N	NAD	N	N/A	Y	5 out of 9	55.5%
Wong [29]	Case Report	Level IV	Y	Y		1	Y	N/A	Y	Y	Y	N/A	NAD	N	N	Y	N/A	Y	8 out of 10	80%

The predominant methodological concerns between the included studies were: lack of justification of the sample size (one study did a power calculation [24]), lack of detailed descriptions regarding intervention (six studies utilised multiple interventions and provided inadequate details of the same) [22, 25–29], lack of randomisation (only one study randomised participants [24]), lack of reporting statistical significance (six of the eight studies included statistical significance [22–26, 28]) and lack of reporting clinical significance (three studies reported clinical significance [22,26,29], with one reporting effect size [26]). Additionally, three studies failed to detail ethics approval and participants’ consent [22,28–29].

### Study characteristics

Table 2 provides a summary of the study characteristics 2. A variety of research designs were identified for this systematic review; which included a range of participants from differing countries. The studies were conducted in the United States of America [22, 24, 29], Turkey [25], Sweden [23], Chile [26], New Zealand [28] and India [27]; published between 2010–2017. All studies investigated the effects of physiotherapy interventions on PPSP in relation to a range of outcome measures. Despite having the same patient population, there was a large distribution of the duration of PPSP ranging from three months to seven years.

**Table 2:** Study characteristics.

Study	Country	Surgery	Time Since Surgery	Sample Size	Mean age	Gender	Study intervention	Co-intervention	Comparator/ Control	Outcome Addressed	Follow up
Albayrak [25]	Turkey	Total Knee Arthroplasty	>38 months	Intervention: 17	Intervention: 65.8 (+/- 6.5)	Intervention: F; 15, M: 2	PRF + TENS + Exercise	Nil	TENS + Exercise	Pain- VAS	15 days, 1 month and last control examination
		
				Comparator: 22	Comparator: 62.1 (+/- 4.9)	Comparator: F: 20, M: 2					
Bailey [22]	USA	Laminectomy	>6 months	43	64	F: 26 M:17	Physical rehab + CBT	Medications	Nil	"Pain: NRS, pain self-efficacy Depression: MPI, PSEQ, CES-D, SF36 "	3 weeks
Bjersa [23]	Sweden	Thoraco-abdominal oesophagus resection	2–7 years	8	61.9 (range 51–67)	F:3 M:5	Osteopathic manual therapy	Medications	Nil	Pain- BPI-SF	10 weeks
Nunez-Cortes [26]	Chile	Total Knee Arthroscopy	>3 months	14	66.8 (+/-8.1)	F:11 M:3	Stretching, stabilisation, strength training exercises and dry needling	Nil	Nil	Pain- VAS, WOMAC	4 weeks
Pfitser [24]	USA	Neck Dissection	Intervention: 39 (29–48)	Intervention: 28	Intervention: 61 (54–68)	Intervention: F:13, M:15	Acupuncture	Medications	Usual Care	"Pain-NRS, MCM"	10 days before, 3 days before, weekly for 5 weeks, then at 35 days and 42 days
			
			Comparator: 34 (15–44)	Comparator: 30	Comparator: 57 (50–63)	Comparator: F: 7 M:23					
Pons [28]	New Zealand	Multilevel spinal fusion for spinal stenosis	10 months	1	68	M:1	TENS + Exercise	Medications	Nil	Pain-NRS	15 weeks
Vas [27]	India	Total Knee Replacement	"1 year 4 months"	2	74	F:1 M:1	PRF, dry needling, physiotherapy, medication	Nil	Nil	Pain-NRS	Day 1, day 15, 1 month, 3 months, 6 months
Wong [29]	USA	laparoscopic appendectomy with a right ovarian cystectomy	1 Year	1	28	F:1	Manual therapy, therapeutic exercises, home exercise program	Nil	Nil	Pain- VAS	"Initial examination Re-examination (7 sessions, 3 wk) Discharge (10 sessions, 7 wk)"

### Participant characteristics

There was a combined total of 166 participants, with the number in each study ranging from one to 58, aged between 28 and 74 years. Of total participants, there were more female (97) participants than male (69). Ethnicity of participants was reported in one study [22]. Preceding surgical procedures differed between studies, which included total knee arthroplasty [25–27], laminectomy [22], thoracoabdominal oesophagus resection [23], multi-level lumbar fusion [28] and abdominal and pelvic adhesion [29].

### Intervention type

Despite all studies investigating physiotherapy management of PPSP, there was perceptible variability in both the physiotherapy interventions used and how they were used. Six of the eight studies used multiple interventions [22,25–29], with only two studies using a single intervention [23–24], making it difficult to determine causality. Interventions included electrotherapy, such as transcutaneous electric nerve stimulation (TENS) and Pulsed Radio-Frequency (PRF) [25, 27–28]; manual therapy, such as massage; osteopathy and passive movements [23, 27–29]. Seven studies included exercise as an intervention [22–23, 25–28] and four studies used emerging techniques as an intervention, including acupuncture and dry needling [24, 26–27, 29].

### Outcome measures (OMs)

The types of outcomes and OMs varied between studies to evaluate the effectiveness of physiotherapy interventions. Studies utilised a combination of subjective measures: including pain; QoL and depression; and objective measures, such as the timed up and go test (TUG), the six-minute walk test (6MWT) and the 30-second chair stand test (30-CST). The time point measurements differed between studies with one measuring the immediate effect (same day) [29], and all eight-measuring the medium-term effects (one month to four months) [22–29]. No studies measured, nor reported, adverse outcomes because of physiotherapy. The range of outcome domains and measures used for each study are identified in Table 3.

**Table 3:** Outcome measures.

Study	Outcome Domain and Outcome Measures																
	Pain											QOL		Physical Function		Depression	
	VAS	NRS	PSEQ	MPI-LC	MPI-I	BPI-SF	WOMAC-Pain	PCS	OKS	CMS	M-CMS	WOMAC	SF-36	6MWT	TUG	PHQ-9	CES-D
**Albayrak [25]**	✓											✓					
**Bailey [22]**		✓	✓	✓	✓			✓					✓	✓			✓
**Bjersa [23]**						✓											
**Nunez-Cortes [26]**	✓						✓							✓	✓		
**Pfitser [24]**		✓								✓	✓						
**Pons [28]**		✓															
**Vas [27]**		✓							✓							✓	
**Wong [29]**	✓																

ABBREVIATIONS: QOL = Quality of life OKS: oxford knee scale, PHQ-9 = Patient health questionnaire, CMS = Constant-Murley Score, M-CMS = modified Constant-Murley score, PSEQ = Pain Self-Efficacy Scale Questionnaire, MPI = Multidimensional Pain Inventory (LC- life control, I- interference), SF-36 = Short Form (36) Health Survey, CES-D = Centre for Epidemiologic Studies Depression Scale, PCS = Pain Catastrophising Scale, BPI-SF = Brief Pain Inventory- Short Form, WOMAC = The Western Ontario and McMaster Universities Osteoarthritis Index, OKS = Oxford Knee Score, VAS = visual analogue scale, NRS = Numerical rating scale

### Pain

All eight studies measured pain using several different OMs and reported positive impacts of physiotherapy interventions on pain [22–29]. However, four of these studies supported this positive impact with statistically significant findings (p<0.05) [22, 24–26], while the remaining four did not [23, 27–29]. The OMs utilised to measure pain included the Visual analogue scale (VAS) [25–26], Numerical Rating Scale (NRS) [22,24], Pain Self-Efficacy Scale Questionnaire (PSEQ) [22], Multidimensional Pain Inventory (MPI) [22], The Western Ontario and McMaster Universities Osteoarthritis Index (WOMAC) [26], Pain Catastrophizing Scale (PCS) [22], Constant-Murley Score (CMS) [24] and the Modified Constant Murley Score (M-CMS) [24].

### QOL

Two studies measured the effects of physiotherapy management on QOL using self-administered questionnaires, including the WOMAC and the 36-item short form survey (SF-36) [22, 25]. Studies by Albayrak and colleagues [25] provided a package of care to participants, comprising of: TENS, exercise and PRF; as well as cognitive behavioural therapy, exercise and education [22]. When using WOMAC, Albayrak and colleagues [25] reported a significant (p<0.05) reduction in total scores between pre-treatment, 15 days post-treatment and one-month post-treatment; between groups. Bailey and colleagues [22] also discovered a statistically significant difference between admission and discharge scores on the SF-36 (p<0.05).

### Physical function

Two studies measured the effect on physical function and utilised the 6MWT, the TUG and the 30-CTS to measure the same [22, 26]. Similarly, Nunez-Cortes and colleagues [26] used a package of care in the management of the participants, comprising of dry needling and exercise. Bailey and colleagues [22] used the 6MWT to assess physical function and found a statistically significant improvement (p<0.05) between admission and discharge; however, participants were still below normative values for their age group. Nunez-Cortes and colleagues [26] used the 6MWT, TUG and 30-CST and found a statistically significant improvement (p<0.05) in the 6MWT pre- and post-intervention. However, they found no statistically significant improvement in the TUG and 30-CST scores [26].

### Depression

Two studies measured changes in depression symptoms and utilised the Centre for Epidemiologic Studies Depression Scale (CES-D) and the Patient Health Questionnaire-9 (PHQ-9) [22, 27]. Vas and colleagues [27] provided a package of care in managing the participants, comprising of: PRF, TENS, dry needling and exercises. They reported a positive impact in the PHQ-9 scores; however, did not report statistical significance. Bailey and colleagues [22] used the CES-D and reported a positive statistical significance (p<0.05) between pre- and post- discharge, indicating a reduction in depressive symptoms and negative pain-related cognition.

### Summary of results

The results from the included studies, which highlights the range of outcomes measured (16 different outcomes) within four domains, are summarised in Table 4. Irrespective of this, collective findings indicate that physiotherapy management has a positive impact across all domains. There is consistent evidence to suggest that physiotherapy had a positive impact in symptom reduction (pain), which is the most commonly measured outcome. While QoL, physical function and depression were measured by only a handful of studies (two studies each for QoL and depression and three studies for physical function), available evidence points to a positive impact from physiotherapy. Regarding physical function, two studies reported statistically significant improvements in the 6MWT [22, 26] and one study reported improvement in the TUG and 30-CST [26]. While these are encouraging findings, given the small number of studies, especially for outcome domains such as QoL, physical function and depression, caution is required when interpreting these results.

**Table 4:** Summary of results.

Study	Effect of physiotherapy interventions for the management of adults with PPSP																
	Pain											QOL		Physical Function		Depression	
	VAS	NRS	PSEQ	MPI-LC	MPI-I	BPI-SF	WOMAC-Pain	PCS	OKS	CMS	M-CMS	WOMAC	SF-36	6MWT	TUG	PHQ-9	CES-D
**Albayrak [25]**	↓↓ (+)*											↑(+)*					
**Bailey [22]**		↓ (+)*	↑(+)*	↑(+)*	↓(+)*			↓(+)*					↑(+)*	↑(+)*			↓(+)*
**Bjersa [23]**						↓(+)?											
**Nunez-Cortes [26]**	↓ (+)*						↑(+)*							↑(+)*	↓(+)*		
**Pfitser [24]**		↓(+)*								↑(+)*	↑ (+)*						
**Pons [28]**		↓(+)?															
**Vas [27]**		↓(+)?							↑(+)?							↓(+)?	
**Wong [29]**	↓ (+)?																

Notes: ↑ = increased, ↓ = decreased, (+) = positive change/ improvement, (-) = negative Change

* = statistical significance (p <0.5), (?) = significance not reported

### NHMRC FORM framework

The synthesis of results using the NHMRC FORM framework is shown in Table 5. Despite positive outcomes reported in some studies, especially the positive impact of physiotherapy for pain, methodological concerns of the evidence base lowered the overall grade of recommendation. While these findings may be supportive of physiotherapy, caution is required when implementing these recommendations.

**Table 5:** NHMRC FORM framework.

Component	Grade	Comments
**1. Evidence Base **	D-Poor *Level IV studies*, *or level I to III studies with high risk of bias *	Quantity: 8 studies Participants: 166 adults with PPSP Level II: 1 study Level III-2: 1 study Level III-3: 2 studies Level IV: 4 studies
**2. Consistency **	C—Satisfactory *Some inconsistency reflecting genuine uncertainty around clinical question*	Findings consistent Multiple study designs Statistical significance reported in 4/8 studies Heterogeneous interventions Varied population- age, surgery type Varied outcome measures and time point measurements
**3. Clinical impact **	D–Poor *Slight or poor*	Consistent findings for primary and secondary outcomes: pain, QoL, physical function and depression No adverse effects reported Intervention protocol adequately described however, no justification for parameters or development process was outlined
**4. Generalisability **	B-Good *Population(s) studied in body of evidence are similar to the target population *	Population of studies is similar to the target population Age range: 28–74 years The current evidence base lacks clarity in terms of reporting of comorbidities and co-interventions and its impact on outcomes Studies conducted in six different countries that have different health care contexts
**Grade of recommendations **	D–Poor Body of evidence is weak, and recommendation should be applied with caution	Overall, these studies were low level of evidence and were of moderate methodological quality. While there were generally consistent positive findings, the current evidence base lacks uniformity with regards to participant characteristics, varied time periods since diagnosis, interventions delivered, and its parameters and outcomes measured for adults with PPSP.

## Discussion

This systematic review aimed to address the evidence in the emerging area of physiotherapy management of PPSP. Previous systematic reviews have addressed the effectiveness of pharmaceutical management, which have resulted in equivocal recommendations. There was a modest evidence base, consisting of eight studies representing varying research designs. The summarised findings from this review indicate that physiotherapy interventions are likely to have a positive impact across a range of outcomes including pain, QoL, physical function and depression. This was particularly evident for pain, which indicates that physiotherapy interventions are likely to be effective in the management of adults with PPSP. Despite these positive findings, caution is required when interpreting these findings due to the methodological issues within, and lack of uniformity of, the evidence base. As this is the first systematic review to map the literature on the effectiveness of physiotherapy interventions for adults with PPSP, it showcases where there is consistent evidence (pain), emergent evidence (QoL, physical function and depression) and areas for future research.

### Effect of physiotherapy interventions on various outcomes

The use of physiotherapy interventions for managing PPSP identified positive effects across several outcome domains including subjective and objective measures, highlighting the potential role of physiotherapy for these patients. The findings of this systematic review are supported by previously published research on the positive effects of physiotherapy for persistent pain. For example, physiotherapy interventions have been shown to reduce pain across a range of conditions through diverse interventions [36]. This includes patient population which are similar to PPSP such complex regional pain syndrome [37]. As reported in this systematic review, physiotherapy interventions are often offered as a package of care which in turn may address diverse and multifactorial components which underpin PPSP.

Two studies reported improvements in depression in patients with PPSP [22, 27]. These positive results are likely as physiotherapy management often includes exercise prescription, which has previously shown to improve anxiety and depression symptoms for a range of conditions, including breast cancer and spinal disorders [38–39]. Exercise has previously been shown to be effective in improving QoL and psychological functioning [40] as well. Furthermore, in the general adult population, higher levels of exercise are associated with decreased symptoms of anxiety and depression, in addition to an overall increased health related QoL [41–43]. The benefits exercise has on depression and anxiety may occur due to reducing cortisol levels, increasing a patient’s self-esteem and acting as a diversion for negative thoughts [43]. In addition, physiotherapists often incorporate group classes when exercise is included in the management of patients and this social contact may be beneficial in reducing the above symptoms [43]. It has been suggested that physiotherapy should be considered an adjunct to antidepressants and/or psychotherapy in moderate to severe depression [44].

Two studies also reported statistically significant improvement in QoL after physiotherapy management of PPSP [22, 25]. As PPSP is associated with hospital stays, physiotherapy has been shown to effective in improving QoL in other patient population, such has post-cancer treatment or intensive care unit stays, who have also experienced hospital stays [45–46]. One possible explanation for these findings is that hospital-based physiotherapy may better prepare patients to transition into the community and subsequently by focussing on improved physical activity, function and decreasing troublesome symptoms, physiotherapy may be effective in improving QoL [45]. Similarly, two studies [22, 26] reported statistically significant improvements in physical function by increasing the distance walked in the 6MWT and decreasing time taken to perform the TUG. These findings are supported by previous research which has demonstrated that exercise programs are beneficial in increasing physical function in frail older adults by increasing gait speed, improving balance and activities of daily living [47]. Physiotherapy may improve physical functioning by addressing psychological factors (through education), deconditioning (through exercises) and pain (through a package of care consisting of education, manual therapy and exercises).

### Limitations

This systematic review was underpinned by best practice in the conduct of systematic reviews (PRISMA). However, as with any research, there are limitations to this systematic review as well. In order to avoid publication bias, grey literature and secondary searching strategies were implemented. However, unlike black literature search, which can be standardised and applied across multiple databases, grey literature searching is complex and imprecise. Therefore, some publications may have been missed. Due to access and resource limitations, only publications in English language were included and hence language bias should be acknowledged. While this systematic review utilised current and universally agreed definition of PPSP as part of its literature selection process, there may have been other studies which were excluded as they utilised other definitions of PPSP. By using the current and universally agreed definition of PPSP, this systematic review was able to compare similar evidence.

While the systematic searching of the literature identified a modest body of evidence with consistent positive findings, there was some concerns with the methodological quality of the included studies. The sample sizes were generally small and lacking justification, possibly due to the fact that many of the research studies were low quality studies, for which sample size calculations/justifications may be seen to be unnecessary. Due to the nature of the intervention, blinding of patient and therapist was not possible, although the assessor could be blinded. While placebo and Hawthorne effects may not be avoided, measurement bias could be. Given the nature of physiotherapy intervention, which is often provided as a package of care, in some studies physiotherapy interventions were complemented with other interventions (including medications). While this may reflect what occurs in clinical practice settings, the role of co-intervention bias should be considered. Due the complex nature of pain, there was a variety of outcome measures utilised across the studies. This made direct comparison between the studies difficult. Given that more than half of the included studies were case studies/series, generalisability of the findings to a wider population context is limited. While physiotherapy may play a role in the management of PPSP, access to physiotherapy services is likely to be influenced by a number of factors such as private health insurance, local health care models of service delivery, funding models and requirements. These factors need to be considered when considering physiotherapy as an option in the management of PPSP.

## Conclusion

### Implications for practice

There is an emerging body of evidence to support the use of physiotherapy as a treatment option for adults with PPSP. There were positive impacts across a range of outcomes including pain, QoL, physical function and depression. However, while physiotherapy may be considered in the management of PPSP in adults, it must be recognised that the current evidence base suffers from several methodological concerns and therefore implementation of these recommendations should be made with caution.

### Implications for practice and future research

A modest body of evidence has been identified to support the use of physiotherapy interventions for adult patients with PPSP. However, methodological concerns within the evidence base have also been recognised, highlighting the need for further research. While physiotherapy is commonly offered as a package of care in clinical practice settings, in the research context there is variability in results in terms of intervention parameters. Future research would benefit from studies that focus on developing standardised intervention parameters. Similarly, future research could also add to the current evidence base by developing and implementing standardised outcome measures for PPSP. Finally, methodologically sound randomised controlled trials that are conducted with larger sample sizes, with clear control and intervention groups with randomisation, using power calculations, and include long term follow-up would assist in identifying the sustained impact of physiotherapy on adults with PPSP. This could be complemented with information regarding clinical significance. This will increase generalisability, clinical impact and provide unequivocal evidence for the effectiveness of physiotherapy for adults with PPSP.

## Supporting information

S1 Appendix PRISMA checklist. (DOC)

S2 Appendix PROSPERO protocol. (PDF)

S3 Appendix Medline search strategy. (DOCX)

S4 Appendix Medline search syntax. (TIF)
