# Supplementary material for: The effectiveness of physiotherapy interventions on pain and quality of life in adults with persistent post-surgical pain compared to usual care: A systematic review
Source: PLoS One. 2019 Dec 13;14(12):e0226227. doi: 10.1371/journal.pone.0226227 (PMC6910682; doi:10.1371/journal.pone.0226227)
Supplement: S2 Appendix — (PDF) [file pone.0226227.s002.pdf]

Effectiveness of physiotherapy management on persistent post-surgical pain: a systematic review

Hamish Peberdy, Jenna McIntosh, David Wishart, Henry Pope, Georgia Brown, Aleisha Robinson, Saravana Kumar

Citation

Hamish Peberdy, Jenna McIntosh, David Wishart, Henry Pope, Georgia Brown, Aleisha Robinson, Saravana Kumar. Effectiveness of physiotherapy management on persistent post-surgical pain: a systematic review. PROSPERO 2019 CRD42019129580 Available from:
http://www.crd.york.ac.uk/PROSPERO/display_record.php?ID=CRD42019129580

Review question

The effectiveness of physiotherapy interventions for the management of adults with persistent post-surgical pain compared to usual care.

Searches

The following electronic databases will be searched: MEDLINE Ovid; Embase; Emcare; The Cochrane Library. The references of relevant articles will be searched to identify potential additional articles (pearling). Grey literature searching will include web engine searching such as google and Google Scholar. The electronic database TROVE will also be searched in addition organisational websites related to the topic such as Pain Australia. Language (English) and human only restrictions will also be applied.

Types of study to be included

This review will include all primary research of quantitative research paradigm including randomised controlled trials, clinical trials, case-controls, cohort studies, pilot studies and case reports/case series. This review will exclude secondary research, protocol studies, opinion papers, editorials, conference presentations and research from the qualitative research paradigm.

Condition or domain being studied

The purpose of this systematic review is to evaluate the effectiveness of physiotherapy management on persistent post-surgical pain in regards to changes in pain, health-related quality of life, anxiety and depression. This review will specifically focus on people experiencing persistent post surgical pain greater than two months post surgery.

Participants/population

Inclusions: adults, persistent post surgical pain, humans

Exclusions: children, people post amputation

Intervention(s), exposure(s)

Inclusion: physiotherapy management

Exclusion: non-physiotherapy management

Comparator(s)/control

Usual care

Context

Main outcome(s)

Including but not limited to:
Pain (visual analague scale, numerical rating scale, multidimensional pain inventory, WOMAC);
Quality of life (SF-36, EuroQoL);
Depression (Centre for Epidemiologic Studies Depression Scale (CES-D);
Physical function (6-minute walk test, timed up and go);

Timing and effect measures

See above

Additional outcome(s)

Not applicable

Timing and effect measures

Not applicable

Data extraction (selection and coding)

A customised data extraction form will be developed by the reviewers using key components of the review question including the population, intervention, comparator, outcomes, study type, and the results of the study. The data extraction form will be trialled independently by two reviewers, on two included studies. The results of the data extraction tool will be assessed by the whole review team and facilitator, with any necessary changes to be made to the form before use of the data extraction tool on the remaining studies. Using the data extraction tool, all six reviewers will independently, extract data from each study to ensure reliability and consistency. Reviewers will resolve any disagreements through group discussion, and if a consensus cannot be attained, the facilitator will make the final decision. Information involved with the data extraction will be stored on both password-protected cloud-based storage and password-protected personal computers. The computer program CovidenceTM will be used to manage study records.

Risk of bias (quality) assessment

All included studies will be reviewed and ranked using the ‘intervention category’ of the National Health and Medical Research Council (NHMRC) hierarchy of evidence (National Health and Medical Research Council 2009) by all six reviewers independently. If there is a discrepancy, the group will meet to discuss and rectify as they present. If no resolution can be achieved, the facilitator will be consulted.

To identify the risk of bias of the included studies, a modified McMaster Critical Appraisal tool – quantitative version will be used by reviewers to independently by using a numerical rating scale, where yes = 1 point, and no = 0 points. An overall score for each study will then be produced to compare results between reviewers. The modified McMasters Critical Appraisal Tool will be used as it is a generic tool, suitable for use on all quantitative study designs (Law et al. 1998). As reviewers will be independently implementing the critical appraisal tool, any differences in score will be discussed between reviewers and if a difference in score remains, the facilitator will be contacted to help make the final decision.

Strategy for data synthesis

A meta-analysis is unlikely to be undertaken due to widespread heterogeneity of the literature found during preliminary scoping of the literature. If this is the case, the NHMRC FORM framework (Hillier et al. 2011) will be used to guide data synthesis, incorporating the following 5 key components:

1.Quantity and quality of evidence

2.Consistency of the study results

3.Clinical impact/ effect size

4.Generalisability

5.Applicability to the Australian health care setting- This component will not be used in this systematic review due to its international focus.

An assessment of each component will be completed which will determine the strength of evidence. A subsequent overall recommendation will be generated which will be a composite of the summarised rating from each component. The NHMRC FORM framework has been chosen as it has been widely used in previous SRs (Mortimer, Privopoulos & Kumar 2014; Machotka et al. 2010; Dars et al. 2018) and the

facilitator has extensive experience in its use.

Analysis of subgroups or subsets
None planned

Contact details for further information
Saravana Kumar
Saravana.Kumar@unisa.edu.au

Organisational affiliation of the review
University of South Australia
www.unisa.edu.au

Review team members and their organisational affiliations
Mr Hamish Peberdy. University of South Australia
Miss Jenna McIntosh. University of South Australia
Mr David Wishart. University of South Australia
Mr Henry Pope. University of South Australia
Miss Georgia Brown. University of South Australia
Miss Aleisha Robinson. University of South Australia
Mr Saravana Kumar. University of South Australia

Type and method of review
Intervention, Systematic review

Anticipated or actual start date
22 March 2019

Anticipated completion date
14 June 2019

Funding sources/sponsors
None

Conflicts of interest

Language
English

Country
Australia

Stage of review
Review Ongoing

Subject index terms status
Subject indexing assigned by CRD

Subject index terms
Humans; Pain, Postoperative; Physical Therapy Modalities

Date of registration in PROSPERO
05 June 2019

Date of publication of this version
05 June 2019

Details of any existing review of the same topic by the same authors

Stage of review at time of this submission

Stage	Started	Completed
Preliminary searches	Yes	No
Piloting of the study selection process	No	No
Formal screening of search results against eligibility criteria	No	No
Data extraction	No	No
Risk of bias (quality) assessment	No	No
Data analysis	No	No

Versions

05 June 2019

PROSPERO
This information has been provided by the named contact for this review. CRD has accepted this information in good faith and registered the review in PROSPERO. The registrant confirms that the information supplied for this submission is accurate and complete. CRD bears no responsibility or liability for the content of this registration record, any associated files or external websites.
