# Supplementary material for: The effectiveness of physiotherapy interventions on pain and quality of life in adults with persistent post-surgical pain compared to usual care: A systematic review
Source: PLoS One. 2019 Dec 13;14(12):e0226227. doi: 10.1371/journal.pone.0226227 (PMC6910682; doi:10.1371/journal.pone.0226227)
Supplement: S3 Appendix — (DOCX) [file pone.0226227.s003.docx]

**Medline search strategy**

| 1. Pain, postoperative/ |
| --- |
| 1. (post?surg* or post?operati* or post?thoracot* or post?laparot* or post?mastect* or post?sternot* or after surg* or after operat* or following operat* or following surg*).mp. |
| 1. Chronic pain/ |
| 1. (Chronic pain* or persistent pain* or long?term pain* or on?going pain* or ppp or ppsp or cpsp).mp. |
| 1. Exp physical therapy modalities/ |
| 1. (physical therap* or physio?therap* or physical treat* or physical medicine* or chest wall oscillat* or home physio* or joint mobile* or manual therap* or movement therap* or psycho?therap or educat* or animal?assisted therap* or equine?assisted therap* or hippotherap* or horse?back riding therap* or pet facilitate* or pet therap* or massag* or drainage, postural or postural drainage or electric stimulation therap* or electrotherapy* or electroacupuncture or pulsed radiofrequency treatment or spinal cord stimulation* or transcutaneous electric* or TENS or exercise movement technique* or exercise therap* or breathing exercise* or dance therap* or tai ji* or tai chi* or yoga or endurance train* or motion therap* or passive motion therap* or muscle stretching exercise* or active stretch* or ballistic stretch* or dynamic stretch* or isometric stretch* or passive stretch* or proprioceptive neuromuscular facilitate* or PNF or relaxed stretch* or static stretch* or static?active stretch* or static?passive stretch* or plyometric exercise* or plyometric drill* or plyometric train* or resistance train* or strength train* or weight?bearing exercise program* or weight?bearing strength program* or weight?lifting exercise program* or weight?lifting strengthening program* or extracorpeal shockwave therap* or hydrotherap* or water therap* or aquatic therap* or musculoskeletal manipulat* or manipulation therap* or applied kinesiolo* or orthop?edic manip* or spinal manip* or cervical manip* or lumbar manip* or soft tissue therap* or acupressure or reflexolo* or craniosacral massag* or myofunctional therap*) .mp. |
| 1. 1 or 2 |
| 1. 3 or 4 |
| 1. 5 or 6 |
| 1. 7 and 8 and 9 |
