# Supplementary figures and images for: The effectiveness of physiotherapy interventions on pain and quality of life in adults with persistent post-surgical pain compared to usual care: A systematic review
Source: PLoS One. 2019 Dec 13;14(12):e0226227. doi: 10.1371/journal.pone.0226227 (PMC6910682; doi:10.1371/journal.pone.0226227)

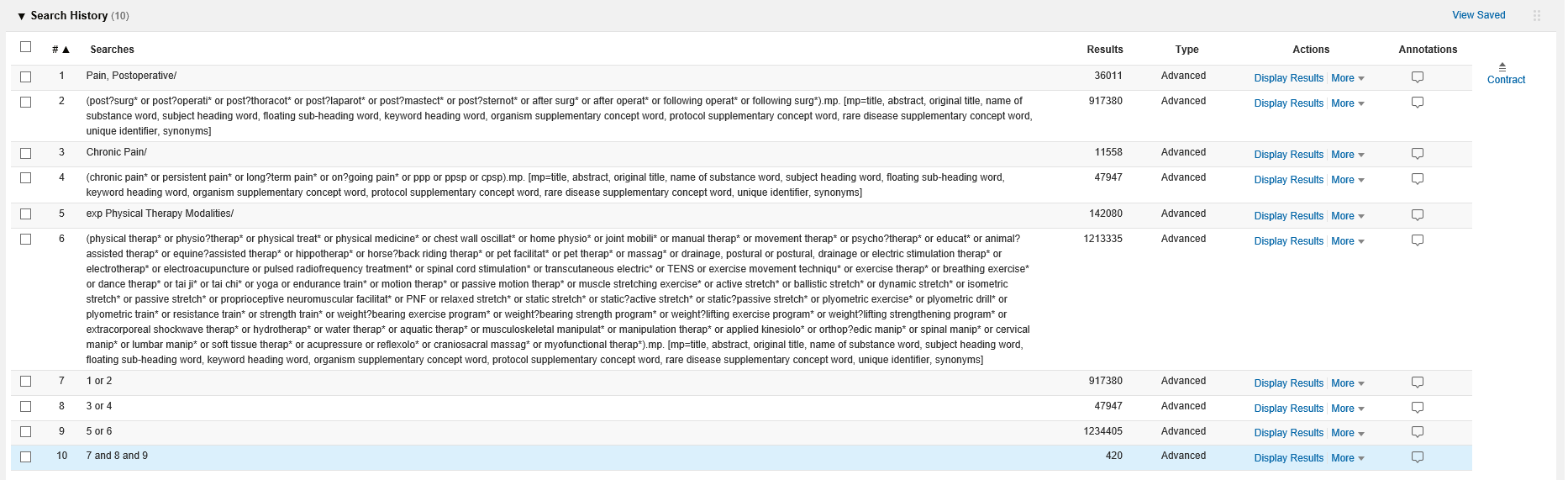

Supplement: S4 Appendix — (TIF) [file pone.0226227.s004.tif]
